# Supplementary material for: 3D sub-diffraction imaging in a conventional confocal configuration by exploiting super-linear emitters
Source: Nat Commun. 2019 Aug 16;10:3695. doi: 10.1038/s41467-019-11603-0 (PMC6697694; doi:10.1038/s41467-019-11603-0)
Supplement: Supplementary file 1 — Supplementary Information [file 41467_2019_11603_MOESM1_ESM.pdf]

Supplementary information: 3D sub-diffraction imaging in a conventional confocal configuration by exploiting super-linear emitters

D. Denkova, M. Ploschner et.al.

Supplementary Note 1: Photo-stability

We studied the photo-stability of the UCNP emitters in two experimentally relevant scenarios. In both cases the particles were located inside the fixed neuronal cells which were used for the uSEE imaging in the paper.

In the first scenario, we scanned the sample through the excitation beam over a period of 5.5 hours at 0.4 mW excitation beam power at the back focal plane of the objective (2.2 mW μm^{-2} peak power density at the sample). This corresponds to imaging in uSEE microscopy mode with resolution two times better than the diffraction limit. We scanned under practically relevant conditions – dwell time of 3 ms, sampling rate of 100×100 pixels, field-of-view (FOV) of $1.67 \times 1.67\,\mu$m. The emission from the particles in the FOV remained relatively stable, as only a minor downward trend in the emission intensity, on the order of 1% per each hour of imaging, was observed (Supplementary Figure 1 **a**).

In the second scenario, we scan at another practically relevant experimental setting, which produces diffraction limited images. This is achieved for excitation power of 2.2 mW at the back focal plane (11.8 mW μm^{-2} peak power density at the sample). We again scanned with the field-of-view of $1.67 \times 1.67\,\mu$m, dwell time of 3 ms and a sampling rate of 100×100 pixels. Again, negligible deterioration of the emission intensity, on the order of 1% per each hour of imaging, was observed as seen in Supplementary Figure 1 **b**, which depicts the emission behaviour over a period of 7 hours.

It is also important to note that the imaging resolution remains the same throughout the whole duration of the imaging – 215 nm for the uSEE imaging regime in Supplementary Figure 1 **a** and 420 nm for the diffraction-limited confocal imaging in Supplementary Figure 1 **b**.

The alignment of the particle with respect to the beam has been verified at regular intervals.

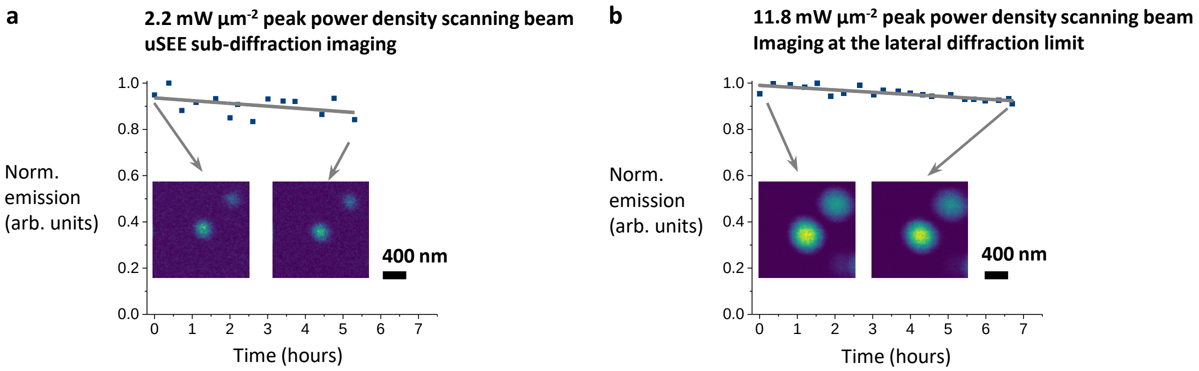

Supplementary Figure 1: **The particle emission intensity decreases with a slow rate of 1% per hour, while the imaging resolution remains stable for practical imaging conditions**. **a** Scanning an excitation beam of peak power density 2.2 mW μm^{-2}, in uSEE microscopy imaging conditions results in negligible drop of the emission intensity of the particle over a period of 5.5 hours. **b** Scanning an excitation beam of peak power density 11.8 mW μm^{-2}, in confocal mode corresponding to diffraction limited resolution, results in negligible decrease of the emission intensity of the particle over a period of 7 hours. In both (a) and (b) the resolution remains stable throughout the measurement.

Supplementary Note 2: Simulating the resolution of the optical system when imaging a non-linear emitter with an arbitrary excitation-emission curve

Section A: General procedure for applying the theoretical framework

In Supplementary Note 2, we detail on the theoretical framework, which we have developed, to numerically calculate the resolution of confocal microscopes employing non-linear emitters with a continually changing slope.

The resolution is determined by the excitation beam shape and by the excitation-emission curve of the emitter. Due to the super-linearity of the emitter, small variations in these factors can lead to significant changes in the resolution. Thus, the excitation beam shape and the excitation-emission curve need to be precisely defined.

We adopt a fully vectorial model to calculate the excitation beam shape B_{ex} (Supplementary Note 2, Section B). The model also accounts for key factors influencing the beam, namely the filling factor of the objective back-pupil and the polarization of the incident light. The resulting expression for the excitation beam shape (Eq. 15) is evaluated numerically in Matlab for the specific parameters of our setup (see Supplementary Note 2, Section F for details).

In addition to the excitation beam shape, a functional form of the excitation vs emission curve U is also needed to predict the resolution. We experimentally determine U_{m}, as described in Supplementary Note 2, Section D and we find a functional form of U by fitting a smoothing spline in Matlab (see Supplementary Note 2, Section E for details).

Finally, equipped with the knowledge of U and the excitation beam shape B_{ex}, we proceed to rule out the effect of the pinhole affecting the resolution in our system. The theory of the detection pathway filtering is presented in Supplementary Note 2, Section C, F along with the conclusion that for our specific experimental conditions, the excitation-emission curve U and the excitation beam shape fully determine the resolution of the system.

Section B: Calculation of the excitation beam shape using fully vectorial approach

We base our calculations on the established approach from Chapter 3 in Ref. [1] to calculate the electric field distribution in the focal region of an aplanatic, high numerical aperture (NA) microscope objective lens of focal length f (Supplementary Figure 2 **a**). In this section, we modify the approach in Ref. [1] to account for the circular polarization of the beam and for the specific filling factor of our microscope objective.

The light source in the form of a linearly polarized (along x'' direction), collimated Gaussian beam with the beam waist w_0 (see Supplementary Figure 2 **a**) can be expressed as

$$\mathbf{E}''_0\left(x'', y''\right) = \begin{bmatrix} 1 \\ 0 \\ 0 \end{bmatrix} \cdot \exp\left(-\frac{x''^2 + y''^2}{w_0^2}\right), \tag{1}$$

where the negligible spreading of the beam along the propagation axis is omitted. Please note that we use (x'', y'') to denote the coordinate system before the dichroic mirror (DM) and (x, y) for the coordinate system after the DM. The $\mathbf{E}''_0$ beam is passed through $\lambda/4$-waveplate with its optical axis at 45° angle with respect to the direction of the incident polarization. Transforming the polarization of the $\mathbf{E}''_0$ beam from linear to circular is essential for the radial symmetry of the beam in the focus of high NA objectives. Using linear polarization leads to highly elliptic beams which results in asymmetric resolution in the lateral plane [1,2]. For this reason, we focus our subsequent analysis on the case of a circularly polarized beam and derive the equations describing the beam in the focus for this situation.

The transformation of the beam $\mathbf{E}''_0$ by $\lambda/4$-waveplate can be mathematically expressed as $\mathbf{E}''_{\mathrm{inc}} = \mathbf{T}_{\lambda/4} \cdot \mathbf{E}''_0$ where

$$\mathbf{T}_{\lambda/4} = \frac{\sqrt{2}}{2} \begin{bmatrix} 1 & i & 0 \\ i & 1 & 0 \\ 0 & 0 & 1 \end{bmatrix}, \tag{2}$$

Applying the transformation $\mathbf{T}_{\lambda/4} \cdot \mathbf{E}''_0$ leads to the following expression for $\mathbf{E}''_{\mathrm{inc}}$

$$\mathbf{E}''_{\mathrm{inc}}\left(x'', y''\right) = \frac{\sqrt{2}}{2} \begin{bmatrix} 1 \\ i \\ 0 \end{bmatrix} \exp\left(-\frac{x''^2 + y''^2}{w_0^2}\right). \tag{3}$$

The circularly polarized beam $\mathbf{E}''_{\mathrm{inc}}$ is then reflected on the dichroic mirror. If we assume that the dichroic mirror reflects the beam in a way that does not change the polarization state, the reflection is simply a coordinate mapping from $(x'', y'') \rightarrow (x, y)$ and the beam incident on the objective lens becomes

$$\mathbf{E}_{\mathrm{inc}}\left(x, y\right) = \frac{\sqrt{2}}{2} \begin{bmatrix} 1 \\ i \\ 0 \end{bmatrix} \exp\left(-\frac{x^2 + y^2}{w_0^2}\right). \tag{4}$$

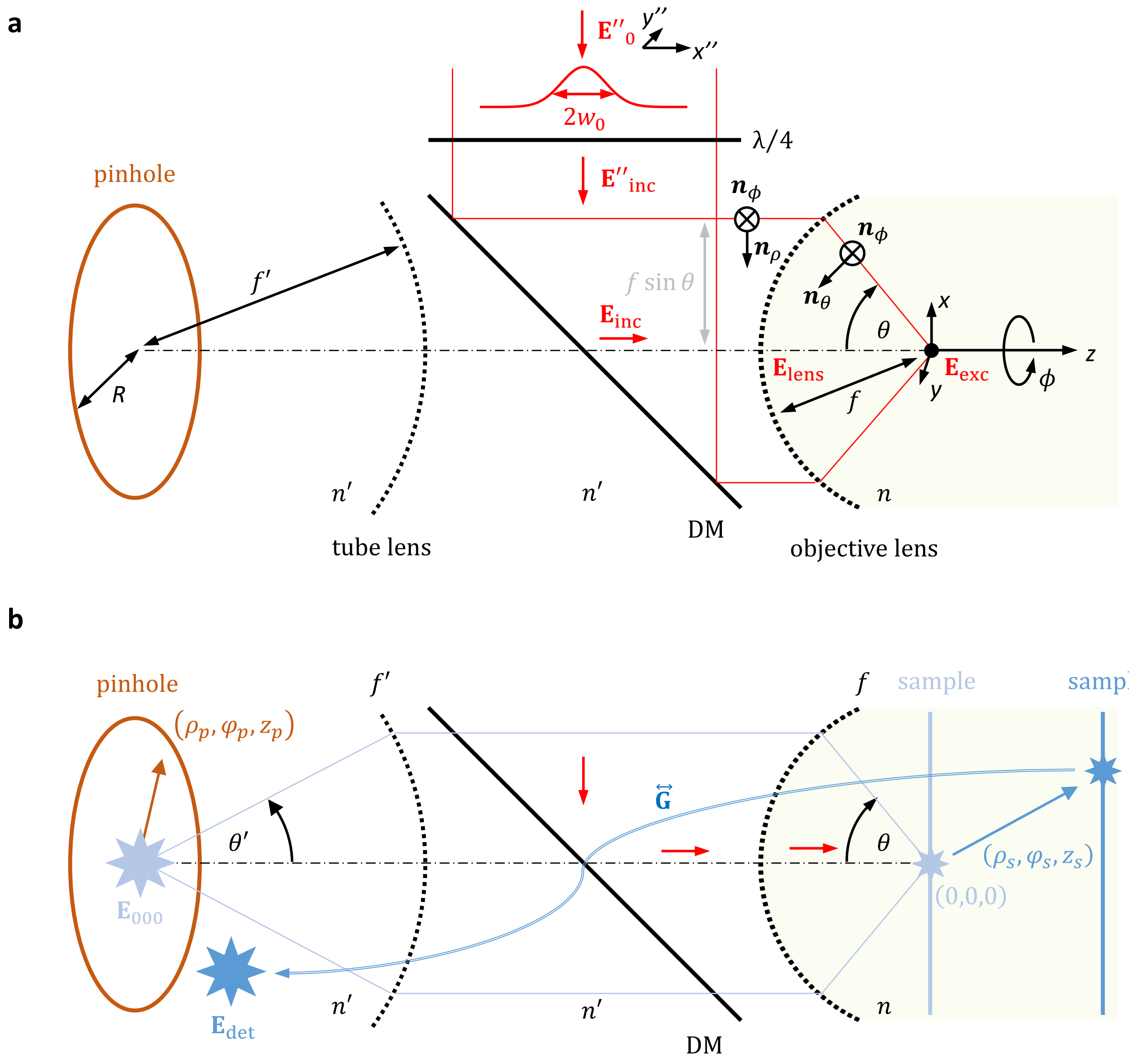

Supplementary Figure 2: **Schematic diagram of a confocal microscope**. In (**a**), we schematically show the geometry, parameters and coordinates used for the derivation of the excitation beam shape in the focus of an objective lens. The pinhole and the tube lens are displayed only for completeness as they have no role on the excitation beam path. In (**b**), we schematically show the formation of the dipole image in the detection plane for a dipole emitter positioned in the origin $(0, 0, 0)$ and displaced by (ρ_s, φ_s, z_s) from the origin.

Here, we take advantage of the sine condition of geometrical optics (see Supplementary Figure 2 **a** and Ref. [1]) which relates the distance of the incident ray from the optical propagation axis (x^2+y^2) and the angle of the corresponding refracted ray θ with respect to the same axis. As per Supplementary Figure 2 **a**, the incident and the refracted rays are related via

$$x^2 + y^2 = f^2 \sin^2 \theta. \tag{5}$$

In order to proceed further, we need to find an expression for $\mathbf{E}_{\text{inc}}$ immediately to the outside of the spherical surface of radius f representing the objective lens. For the lens with a focal length f and after denoting $\mathbf{c} = \frac{\sqrt{2}}{2}[1, i, 0] = \frac{\sqrt{2}}{2}(\mathbf{n}_x + i\mathbf{n}_y + 0\,\mathbf{n}_z)$, with $\mathbf{n}_x$, $\mathbf{n}_y$ and $\mathbf{n}_z$ being the Cartesian coordinates unit vectors, we can express $\mathbf{E}_{\text{inc}}$ in the spherical coordinates (f, θ, ϕ) with the origin in the focus of the aplanatic lens as

$$\mathbf{E}_{\text{inc}}(f, \theta, \phi) = \exp\left(-\frac{f^2 \sin^2 \theta}{w_0^2}\right)\left[(\mathbf{c} \cdot \mathbf{n}_\phi)\,\mathbf{n}_\phi + (\mathbf{c} \cdot \mathbf{n}_\rho)\,\mathbf{n}_\rho\right]. \tag{6}$$

Here, we have used the sine condition of geometrical optics $x^2 + y^2 = f^2 \sin^2 \theta$ and projected $\mathbf{E}_{\text{inc}}$ into vector

components

$$\begin{aligned}\mathbf{n}_\rho &= \cos\phi\,\mathbf{n}_x + \sin\phi\,\mathbf{n}_y \\ \mathbf{n}_\phi &= -\sin\phi\,\mathbf{n}_x + \cos\phi\,\mathbf{n}_y\end{aligned} \tag{7}$$

as seen in Supplementary Figure 2 **a**.

To find the expression of the field at the inside of the spherical surface we need to consider that the energy carried by the incident and the refracted ray must be the same. To satisfy this condition, the amplitude of the field at the inside of the lens has to be multiplied by a factor of $\sqrt{\frac{n'}{n}}\sqrt{\cos\theta}$, where n' is the refractive index to the outside of the lens and n is the refractive index on the inside of the lens, respectively (see Ref. [1] for derivation of this condition). Here, we assumed that the permeability of the inside/outside media is equal to 1, which is a valid assumption for optical elements used in microscopes. We further assumed that the Fresnel transmission coefficients along $\mathbf{n}_\rho$ and $\mathbf{n}_\phi$ are equal, which is a very good approximation for an air-glass interface of a lens. Finally, as seen in Supplementary Figure 2 **a**, the spherical surface does the following mapping of the vectors (transition from outside to inside)

$$\begin{aligned}\mathbf{n}_\phi &\rightarrow \mathbf{n}_\phi \\ \mathbf{n}_\rho &\rightarrow \mathbf{n}_\theta,\end{aligned} \tag{8}$$

where

$$\mathbf{n}_\theta = \cos\theta\cos\phi\,\mathbf{n}_x + \cos\theta\sin\phi\,\mathbf{n}_y - \sin\theta\,\mathbf{n}_z. \tag{9}$$

After taking the above into consideration, we can write the field immediately to the inside of the lens as:

$$\mathbf{E}_{\text{lens}}\left(f,\theta,\phi\right) = \sqrt{\frac{n'}{n}}\sqrt{\cos\theta}\exp\left(-\frac{f^2\sin^2\theta}{w_0^2}\right)\left[\left(\mathbf{c}\cdot\mathbf{n}_\phi\right)\mathbf{n}_\phi + \left(\mathbf{c}\cdot\mathbf{n}_\rho\right)\mathbf{n}_\theta\right]. \tag{10}$$

Note that we have not only expressed $\mathbf{E}_{\text{lens}}$ on the spherical coordinate grid (f,θ,ϕ), but we have also transformed the vector components of $\mathbf{E}_{\text{lens}}$ from cartesian to spherical coordinate system, centred in the focus of the aplanatic lens.

Substituting from Eq. (7, 9) into Eq. (10) leads to

$$\mathbf{E}_{\text{lens}}\left(f,\theta,\phi\right) = \frac{1}{2}\sqrt{\frac{n'}{n}}\sqrt{\cos\theta}\exp\left(-\frac{f^2\sin^2\theta}{w_0^2}\right)\begin{bmatrix}(1+\cos\theta)-(1-\cos\theta)\cos 2\phi - i\,(1-\cos\theta)\sin 2\phi \\ i\,(1+\cos\theta)+(1-\cos\theta)\cos 2\phi - (1-\cos\theta)\sin 2\phi \\ -2\sin\theta\left(\cos\phi + i\sin\phi\right)\end{bmatrix}. \tag{11}$$

Note that this final form of $\mathbf{E}_{\text{lens}}$ is a vector field dependent on the spherical coordinate grid but with the vector components along the Cartesian directions $(\mathbf{n}_x, \mathbf{n}_y, \mathbf{n}_z)$.

We now modify the expression to account for the filling factor f_0 of the back-aperture of the microscope objective lens, which influences both the lateral and the axial extend of the excitation beam shape. Large filing factors take advantage of the full NA of the microscope objective whereas small filing factors effectively decrease NA and therefore also decrease the resolution of the system. As the axial resolution improves roughly with the square of NA and lateral resolution improves linearly with NA, the filing factor can play a major role in the precise determination of the beam shape and the resolution of the microscope, respectively.

The aperture radius of the lens is given by $f\sin\theta_{\max}$, where $\theta_{\max}$ is the maximum acceptance angle of the cone of light entering the lens. The filling factor f_0 is defined as the ratio between the beam waist and the radius of the back-aperture of the lens $f_0 = w_0/f\sin\theta_{\max}$. Inserting this into Eq. (11) leads to:

$$\mathbf{E}_{\text{lens}}\left(f,\theta,\phi\right) = \frac{1}{2}\sqrt{\frac{n'}{n}}\sqrt{\cos\theta}\,f_w(\theta)\begin{bmatrix}(1+\cos\theta)-(1-\cos\theta)\cos 2\phi - i\,(1-\cos\theta)\sin 2\phi \\ i\,(1+\cos\theta)+(1-\cos\theta)\cos 2\phi - (1-\cos\theta)\sin 2\phi \\ -2\sin\theta\left(\cos\phi + i\sin\phi\right),\end{bmatrix}. \tag{12}$$

where we defined the apodization function of the lens as

$$f_w(\theta) = \exp\left(-\frac{\sin^2\theta}{f_0^2\sin^2\theta_{\max}}\right). \tag{13}$$

The field $\mathbf{E}_{\text{lens}}$, just after the lens interface, is an angular spectrum representation of the field in the focus $\mathbf{E}_{\text{exc}}$, or in other words, $\mathbf{E}_{\text{lens}}$ is an angular spectrum representation of the excitation beam $\mathbf{E}_{\text{exc}}$. The two fields are related

via the Debye-Wolf diffraction integral[1]:

$$\mathbf{E}_{\text{exc}}(\rho,\varphi,z) = \frac{ikf\,\mathrm{e}^{-ikf}}{2\pi} \int_0^{\theta_{\max}} \int_0^{2\pi} \mathbf{E}_{\text{lens}}(f,\theta,\phi)\,\mathrm{e}^{ikz\cos\theta}\,\mathrm{e}^{ik\rho\sin\theta\cos(\phi-\varphi)}\,\sin\theta\,\mathrm{d}\phi\,\mathrm{d}\theta, \tag{14}$$

where the origin of the cylindrical coordinates (ρ,φ,z) is in the focus of the aplanatic lens and k is the wave-number in the lens medium given by $k = 2\pi n/\lambda_0$ (λ_0 is the vacuum wavelength). After performing integration over ϕ, the focal field can be written as:

$$\mathbf{E}_{\text{exc}}(\rho,\varphi,z) = \begin{bmatrix} E^x_{\text{exc}}(\rho,\varphi,z) \\ E^y_{\text{exc}}(\rho,\varphi,z) \\ E^z_{\text{exc}}(\rho,\varphi,z) \end{bmatrix} = \frac{ikf}{2}\sqrt{\frac{n'}{n}}\mathrm{e}^{-ikf} \begin{bmatrix} I_0 + I_2(\cos 2\varphi + i\sin 2\varphi) \\ iI_0 + I_2(\sin 2\varphi - i\cos 2\varphi) \\ -2iI_1(\cos\varphi + i\sin\varphi) \end{bmatrix}, \tag{15}$$

where the functions I_0, I_1, I_2 are given by

$$\begin{aligned} I_0(\rho,z) &= \int_0^{\theta_{\max}} f_w(\theta)\sqrt{\cos\theta}\sin\theta\,(1+\cos\theta)\,J_0(k\rho\sin\theta)\,\mathrm{e}^{ikz\cos\theta}\,\mathrm{d}\theta \\ I_1(\rho,z) &= \int_0^{\theta_{\max}} f_w(\theta)\sqrt{\cos\theta}\sin^2\theta J_1(k\rho\sin\theta)\,\mathrm{e}^{ikz\cos\theta}\,\mathrm{d}\theta \\ I_2(\rho,z) &= \int_0^{\theta_{\max}} f_w(\theta)\sqrt{\cos\theta}\sin\theta\,(1-\cos\theta)\,J_2(k\rho\sin\theta)\,\mathrm{e}^{ikz\cos\theta}\,\mathrm{d}\theta, \end{aligned} \tag{16}$$

where J_n is the nth-order Bessel function of the first kind. The integrals can be evaluated numerically to yield the excitation beam shape $B_{\text{ex}}(\rho,\varphi,z) = |\mathbf{E}_{\text{exc}}|^2$ in the focus of a high NA microscope objective. We do this in the specific case of our setup in Supplementary Note 2, Section F.

Section C: Calculating the field in the detector plane

The precise knowledge of the excitation beam shape allows us to describe how strongly the particles get excited within each position of the beam (Supplementary Figure 2 **b**). In the following we assume that the $\mathbf{E}_{\text{exc}}(\rho,\varphi,z)$ induces an emitting dipole (emitter assumed infinitesimally small), with the dipole moment $\mathbf{p}'$ aligned with the electric field of the excitation $\mathbf{E}_{\text{exc}}$:

$$\mathbf{p}'(\rho,\varphi,z) = \begin{bmatrix} p'_x(\rho,\varphi,z) \\ p'_y(\rho,\varphi,z) \\ p'_z(\rho,\varphi,z) \end{bmatrix} = \frac{\alpha}{|\mathbf{E}_{\text{exc}}(\rho,\varphi,z)|} \begin{bmatrix} E^x_{\text{exc}}(\rho,\varphi,z) \\ E^y_{\text{exc}}(\rho,\varphi,z) \\ E^z_{\text{exc}}(\rho,\varphi,z) \end{bmatrix} \tag{17}$$

where α is the polarisability. Here, we normalized the magnitude of the dipole moment $\mathbf{p}'$ with respect to the excitation intensity $|\mathbf{E}_{\text{exc}}|$, as we need to introduce the magnitude of the dipole emission at the later stage for the special case of a nonlinear emitter. The magnitude of the dipole emission depends on the excitation vs emission curve U, which encompasses the material interaction of the light with the particle. Therefore, we hypothesize that the amplitude of the radiating electric dipole moment for a case of a nonlinear emitter, having a continually changing slope, can be written as:

$$A \propto \sqrt{U\left(|\mathbf{E}_{\text{exc}}(\rho,\varphi,z)|^2\right)} \tag{18}$$

and the nonlinear radiating dipole expression, with the emission strength taken into account, becomes

$$\mathbf{p}(\rho,\varphi,z) = \frac{\alpha\sqrt{U\left(|\mathbf{E}_{\text{exc}}(\rho,\varphi,z)|^2\right)}}{|\mathbf{E}_{\text{exc}}(\rho,\varphi,z)|} \begin{bmatrix} E^x_{\text{exc}}(\rho,\varphi,z) \\ E^y_{\text{exc}}(\rho,\varphi,z) \\ E^z_{\text{exc}}(\rho,\varphi,z) \end{bmatrix}. \tag{19}$$

The magnitude of the induced dipole moment and its orientation allows us to back-propagate the light emitted by the dipole though a microscope objective, and subsequently focus the light by a tube lens onto a detector plane.

The electric field in the detector space $\mathbf{E}_{000}(\rho_d, \varphi_d, z_d)$ due to a radiating dipole in the focus of the aplanatic lens $(0,0,0)$ (see Supplementary Figure 2 **b**) can be readily calculated using the dyadic Green's function $\overleftrightarrow{\mathbf{G}}_0(\mathbf{r},\mathbf{r}_0)$ approach. The electric field $\mathbf{E}_{000}(\mathbf{r})$ from a dipole $\mathbf{p}$ positioned at $\mathbf{r}_0 = (0,0,0)$ can be found via[1]:

$$\mathbf{E}_{000}(\mathbf{r}) = \frac{\omega^2}{\epsilon_0 c^2}\overleftrightarrow{\mathbf{G}}_0(\mathbf{r},\mathbf{r}_0)\cdot\mathbf{p}(\mathbf{r}_0). \tag{20}$$

The procedure to find the dyadic Green's function for a case of radiating dipole embedded in medium of refractive index n, with the field collimated with a microscope objective lens of focal length f and focused by a tube lens of a focal length f' onto a detector embedded in medium n' is described in detail in Ref. [1]. For the purpose of brevity, here we only give the final result which is calculated for the assumption of $f' \ll f$ (see Ref. [1] for more details). This assumption is generally valid in high resolution confocal systems with the tube lens having typically two orders of magnitude longer focal length than the microscope objective lens. With the above assumptions, the dyadic Green's function of the radiating dipole along the detection pathway can be written as:

$$\overleftrightarrow{\mathbf{G}}_0(\rho_d,\varphi_d,z_d) = \sqrt{\frac{n}{n'}}\frac{k'}{8\pi i}\frac{f}{f'}\mathrm{e}^{i\left(kf-k'f'\right)}\begin{bmatrix}(K_0+K_2\cos 2\varphi_d) & K_2\sin 2\varphi_d & -2iK_1\cos\varphi_d\\ K_2\sin 2\varphi_d & (K_0-K_2\cos 2\varphi_d) & -2iK_1\sin\varphi_d\\ 0 & 0 & 0\end{bmatrix}, \tag{21}$$

where functions $K_n(\rho_d,\varphi_d,z_d)$ are given by:

$$\begin{aligned}
K_0(\rho_d,z_d) &= \int_0^{\theta_{\max}}\sqrt{\cos\theta}\sin\theta\,(1+\cos\theta)\,J_0\left(k'\rho_d\sin\theta\, f/f'\right)\mathrm{e}^{ik'z_d\left(1-1/2(f/f')^2\sin^2\theta\right)}\,\mathrm{d}\theta\\
K_1(\rho_d,z_d) &= \int_0^{\theta_{\max}}\sqrt{\cos\theta}\sin^2\theta J_1\left(k'\rho_d\sin\theta\, f/f'\right)\mathrm{e}^{ik'z_d\left(1-1/2(f/f')^2\sin^2\theta\right)}\,\mathrm{d}\theta\\
K_2(\rho_d,z_d) &= \int_0^{\theta_{\max}}\sqrt{\cos\theta}\sin\theta\,(1-\cos\theta)\,J_2\left(k'\rho_d\sin\theta\, f/f'\right)\mathrm{e}^{ik'z_d\left(1-1/2(f/f')^2\sin^2\theta\right)}\,\mathrm{d}\theta
\end{aligned} \tag{22}$$

and k' is the wavevector of emission in the detector medium with refractive index n'.

Finally, the electric field in the detector space originating from the radiating dipole in the focus of the aplanatic lens can be written as

$$\mathbf{E}_{000}(\rho_d,\varphi_d,z_d) = \frac{\omega^2}{\epsilon_0 c^2}\overleftrightarrow{\mathbf{G}}(\rho_d,\varphi_d,z_d)\cdot\mathbf{p}(0,0,0), \tag{23}$$

where (ρ_d,φ_d,z_d) are coordinates in the detector space.

The above equation along with Eq. (19) allows us to eliminate $\mathbf{p}$ and express the electric field in the detector plane as a function of the excitation field $\mathbf{E}_{\mathrm{exc}}$, excitation vs emission curve U and the detection pathway Green's function $\overleftrightarrow{\mathbf{G}}(\rho_d,\varphi_d,z_d)$ as

$$\mathbf{E}_{000}(\rho_d,\varphi_d,z_d) = \frac{\omega^2}{\epsilon_0 c^2}\overleftrightarrow{\mathbf{G}}_0(\rho_d,\varphi_d,z_d)\frac{\alpha\sqrt{U\left(|\mathbf{E}_{\mathrm{exc}}(0,0,0)|^2\right)}}{|\mathbf{E}_{\mathrm{exc}}(0,0,0)|}\mathbf{E}_{\mathrm{exc}}(0,0,0). \tag{24}$$

If we displace the dipole from the origin $(0,0,0)$ (the sample with the emitter is experimentally scanned), to a new position given by $\mathbf{r}_s = (\rho_s,\varphi_s,z_s)$ (see Supplementary Figure 2 **b**), the electric field in the image space will transform as:

$$\mathbf{E}_{000}(\rho_d,\varphi_d,z_d) \to \mathbf{E}_{000}\left(\rho_d-\rho_s M, \varphi_d-\varphi_s, z_d - z_s M^2 n'/n\right) \equiv \mathbf{E}_{\mathrm{det}}(\rho_p,\varphi_p,z_p), \tag{25}$$

where

$$M = \frac{n}{n'}\frac{f'}{f} \tag{26}$$

is the transverse magnification of the detection optical pathway and (ρ_p,φ_p,z_p) are coordinates centred on the pinhole. The integrated detected signal through the pinhole due to a radiating dipole in the position (ρ_s,φ_s,z_s) can then be expressed as:

$$S(\rho_s,\varphi_s,z_s) = \frac{\alpha^2\omega^4}{\epsilon_0^2 c^4}\int_0^R\int_0^{2\pi}\left|\overleftrightarrow{\mathbf{G}}(\rho_p,\varphi_p,z_p)\cdot\sqrt{U\left(|\mathbf{E}_{\mathrm{exc}}(\rho_s,\varphi_s,z_s)|^2\right)}\right|^2 r_p\,\mathrm{d}r_p\,\mathrm{d}\varphi_p, \tag{27}$$

where R is the radius of the pinhole. This equation describes the final resolution of the imaging system as registered by the detector for any radius R of the pinhole placed in front of the detector.

When the pinhole diameter is larger than the image of the dipole, where the size of the dipole image is usually expressed in terms of an Airy unit, the above can be simplified to

$$S(\rho_s, \varphi_s, z_s) \propto U\left(|\mathbf{E}_{\mathrm{exc}}(\rho_s, \varphi_s, z_s)|^2\right) := U(B_{\mathrm{ex}}(\rho_s, \varphi_s, z_s)), \tag{28}$$

where $B_{\mathrm{ex}}(\rho_s, \varphi_s, z_s)$ denotes the intensity beam profile of the excitation beam.

In reality, the integrated detected signal S is equal to the emission intensity profile of the particle I_{em} and this equation can be represented as Eq. 1 in the main text.

This approximation must be considered with extreme care for the case of nonlinear emitters as the size of the dipole image in the pinhole plane depends on the excitation vs emission curve U. As a result, the size of the dipole image changes depending on the excitation power. In the saturation regime of U, and especially for the dipole out-of-focus, the size of the dipole's image can become significant and the above approximation might no longer be valid. However, the above approximation is valid for the range of parameters studied in this manuscript (see Supplementary Note 2, Section F for more details).

Section D: Measurement of the excitation vs emission curve U_{m}

The process of experimentally measuring U_{m} is as follows. We first find a spatially isolated particle as described in Supplementary Methods (*STED imaging*) and ensure that the nearby particles do not contribute to the detected particle intensity for the whole excitation range. This is especially important at higher excitation powers where the saturation of U_{m} leads to large particle profiles which can easily lead to overlap of emission intensities of neighbouring particles. We centre the particle in x,y and z by scanning the particle in both the xy and yz planes. Once the particle is at the centre of the field of view, we illuminate the particle with the excitation laser for a period of 10 s and we measure the accumulated counts on the APD over this period. This is repeated for each excitation power of the U_{m} profile. Whenever the system is not actively acquiring emission signal, we place a beam stop in the excitation path to avoid optically induced damage of the particle, which could result in fading of its emission intensity (Supplementary Note 1; Supplementary Methods (*Pre-illumination procedure for UCNPs*)). Extreme care must be taken, especially at higher excitation powers, where the emission fading can affect the curve U_{m} of the particle. After measuring U_{m}, we sweep through the excitation range to double-check that the emission fading was negligible during the course of U_{m} measurement.

We use a $\lambda/2$-waveplate in tandem with the polarising beam splitter cube and a set of neutral density filters (Supplementary Methods (*Setup description*)) to control the excitation power. The secondary pathway of the polarizing beam splitter cube is used for monitoring the power oscillations introduced to the system by the excitation laser (the power oscillation is around 10% due to the imperfect polarization extinction ratio of the polarization maintaining fibre). The active monitoring of such power oscillations is essential due to high nonlinearity slope of material which substantially magnifies any input power oscillation effect and can easily bias the slope measurement of U_{m}. The attenuation factor of each neutral density filter used was measured experimentally for the relevant wavelength (NE20A-B, Thorlabs and NE10A-B, Thorlabs have an attenuation factor of 20.9 and 9.9, respectively). We ensure that the range of excitation powers for each neutral density filter configuration has sufficient overlap with neighbouring neutral density filter ranges to guarantee that the data is not biased due to interchange of optical components. We measure the background counts of the collection system without a particle present in the field of view and subtract this background from U_{m}.

Finally, we independently verify – by taking images of particles at several excitation powers – that the emission values of U_{m} measured on the static particle are identical to the case of the particle being scanned through the beam. The numerical procedure for the extraction of the peak emission intensity from the raw particle profile images is described in detail in Supplementary Methods (*Measuring the resolution of the system for a non-linear emitter)*.

Section E: Spline interpolation U of a measured excitation vs emission curve U_{m}

As Eq. (28) depends on the local beam intensity rather than the excitation power, the raw excitation vs emission curve U_{m} has to be expressed in terms of the peak beam intensity I_{peak} vs emission, where $I_{\mathrm{peak}} = \max\left(|\mathbf{E}_{\mathrm{exc}}(\rho, \varphi, \mathrm{z})|^2\right)$. The total excitation power P_{exc}, as measured in the back-focal plane of the objective, is related to the peak intensity I_{peak} in the focus of the beam as

$$P_{\mathrm{exc}} = \frac{I_{\mathrm{peak}} \int\limits_0^\infty \int\limits_0^{2\pi} |\mathbf{E}_{\mathrm{exc}}(\rho, \varphi, 0)|^2 \rho \, \mathrm{d}\rho \, \mathrm{d}\varphi}{T} = \frac{I_{\mathrm{peak}} c}{T}, \tag{29}$$

where $T = 0.4$ is the transmittance of our microscope objective at the 976 nm wavelength and c is the result of the numerical integration which is found after substituting from Eq. (15). The numerical integration is performed in Matlab over sufficiently large integration limits to properly account for the beam tail contributions to the total value of the integral.

We numerically fit a spline U through the curve U_{m} (expressed as I_{peak} vs emission) in a log-log space using the Matlab function *fit* with optional parameters *'smoothingspline'*, and with the *'smoothingParam'* parameter set to 0.97. The spline interpolation U expresses the relation $I_{\mathrm{em}} = U(I_{\mathrm{peak}})$ in a functional form. The spline U is needed to calculate the resolution of the system from Eq. (28). This is done in Matlab using the function *feval*.

Section F: Numerical determination of the resolution

First, we use Eq. (15), derived in Supplementary Note 2, Section B to numerically calculate the excitation beam profile for $\lambda_0 = 976\,\mathrm{nm}$ excitation wavelength. We approximate our Olympus UPLSAPO 100XO, NA = 1.4, 100x, objective by an aplanatic lens with an effective focal length of $f = 2\,\mathrm{mm}$ and with the back-aperture diameter of $d = 8\,\mathrm{mm}$. The refractive index of the objective lens, immersion oil, coverslip and the mounting medium where the particles are dispersed is set to $n = 1.518$. The beam waist of the collimated, circularly polarized Gaussian beam illuminating the back-focal plane of the objective is measured to be $w_0 = 6\,\mathrm{mm}$. This means that the filling factor of the beam is $f_0 = 1.5$. The numerical integration of Eq. (15) is done in Matlab using the *integrate* function.

The numerically calculated beam shape is subsequently used as an input in the functional form of U, as derived in Supplementary Note 2, Section E to evaluate the resolution of the entire system by using Eq. (28). We note that the spline fit of U for values of emission with SNR < 1 can lead to unphysical oscillations of U that can lead to the unphysical oscillatory character of the simulated emission profile. For this reason, we restrict the emission profile/resolution simulations to scenarios where the peak power of the excitation beam produces a signal above the noise floor, specifically at least 3σ from the noise floor mean value (σ denotes the standard deviation of the noise floor). Such a choice ensures that the unphysical oscillation appears in the tail of the simulated emission profile and therefore does not affect the full-width-at-half-maximum (FWHM) value, respectively resolution.

The transverse magnification M of the detection pathway, as defined by Eq. (26) is $M = 76$ ($f = 2\,\mathrm{mm}$, $f' = 100\,\mathrm{mm}$, $n = 1.518$, $n' = 1$). Considering M, the pinhole diameter of the collection fibre ($105\,\mu\mathrm{m}$) is larger than the effective Airy unit of the detected beam profile in the pinhole plane across the whole range of experimental studied cases. The pinhole used in our system has therefore negligible effect on the simulated resolution.

We verified the negligible effect of the pinhole numerically by scanning the particle through the excitation beam and using Eq. (27). We calculated the resolution of the system for two cases – for a pinhole with a diameter of $105\,\mu\mathrm{m}$ and for a pinhole diameter corresponding to the case of the pinhole fully open (integration range in Eq. (27) was set to 1 mm). Even at the highest excitation powers used experimentally, which lead to large effective Airy units due to the saturation of U, the effect of the pinhole was negligible and basically within the numerical accuracy of the simulation. Similarly, we verified experimentally that there is a sufficient leeway in the pinhole alignment in all three directions such that no change in the detection PSF is observed. The numerical and experimental verification of the negligible effect of the pinhole conclusively demonstrates that the observed resolution improvement in SEE microscopy is purely due to material effects and not a combination of pinhole filtering and material properties.

Supplementary Note 3: Axial resolution vs excitation and vs emission

The axial resolution is simulated from the excitation-emission curves in Figure 4 **a** of the main paper. The analysis of the graphs showing the axial resolution vs emission (Supplementary Figure 3 **a**) and vs excitation (Supplementary Figure 3 **b**) confirm the qualitative observations in section *"Comparison of uSEE microscopy performance with different UCNPs"*) of the main paper regarding the choice of 8% Tm doped UCNPs over 4% Tm doped UCNPs for uSEE microscopy labels. At the same axial resolution, the 8% Tm doped particles have brighter emission (Supplementary Figure 3 **a**). At the same excitation intensity, the 8% Tm offer better axial resolution (Supplementary Figure 3 **b**).

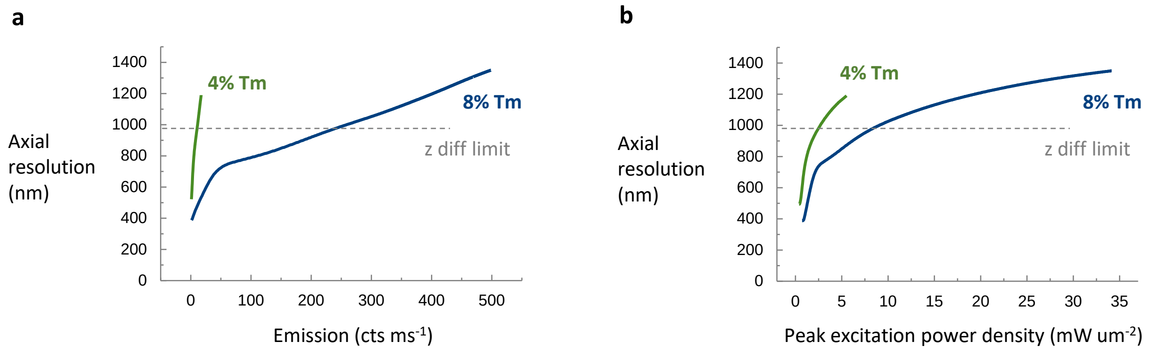

Supplementary Figure 3: **Axial uSEE microscopy resolution across the experimental range a** The axial resolution vs emission graph shows that while imaging at the same axial resolution, the 8% Tm doped particles have brighter emission. **b** The axial resolution vs peak excitation power density graph shows that while imaging at the same excitation power, the 8% Tm doped UCNPs offer better axial resolution. The 4% Tm doped sample is marked in green and the 8% Tm doped sample in blue color. The dashed gray line marks the diffraction limit in the axial direction.

Supplementary Note 4: Comparison of 4% and 8% Tm uSEE images

The 8% Tm doped particles enable better resolution, both in lateral and axial direction, than the 4% Tm doped particles - panels **a** and **b**, respectively in Supplementary Figure 4. The lateral uSEE microscopy resolution is 184 nm for the highly doped UCNPs, and 230 nm for the lower doped nanoparticles. The axial resolution is 390 nm for the 8% Tm sample and 607 nm for the 4% Tm sample. Additionally, the brightness of the 8% Tm is higher, so the images can be obtained faster (8 ms/pxl), compared to the 4% Tm doped sample (20 ms/pxl) and with a better signal-to-noise ratio (12 for the highly doped sample and 5 for the low doped sample).

To facilitate visual comparison between the images in the paper and the Supplementary Information, which are sometimes taken with a different scanning range, we have added a flat background, equal to the average image background, around the experimental images in Supplementary Figure 4. The background is added in the area outside of the dashed black boxes in Supplementary Figure 4. A similar visual aid is used in Figure 1, Figure 2 and Figure 3 in the main text.

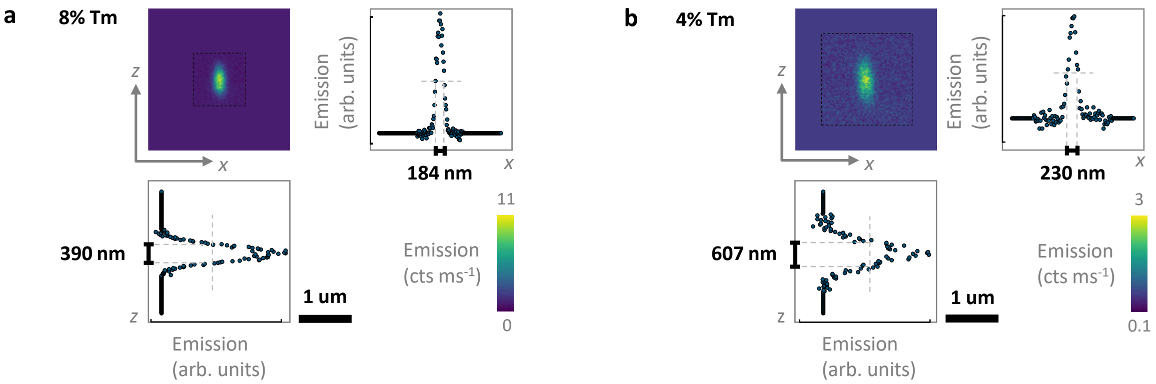

Supplementary Figure 4: **The** 8% Tm **doped particles enable better resolution than the** 4% Tm **doped particles, both in lateral and axial direction.** **a** 8% Tm doped UCNP image with corresponding cross-sections. **b** 4% Tm doped UCNP image with corresponding cross-sections. The blue dots represent the experimental points of the scans.

SUPPLEMENTARY METHODS

Characterization of UCNPs

A TEM picture of the UCNPs is presented in Supplementary Figure 5 **a**.The particles have a shape of hexagonal prisms and they are lying on different facets in the presented Figure. This allows us to determine the average diagonal of the hexagon 45 ± 2 nm and the average height of the prism 47 ± 2 nm.

Spectra of a dense UCNP sample of $NaYF_4$, doped with 20% Yb and 8% Tm, taken at different excitation power are plotted in Supplementary Figure 5 **b**. Note that increasing the power does not simply re-scale the spectrum, but also changes the relative intensity of the peaks due to the complicated balance between different optical processes in the particles – non-radiative energy transfer between the Yb^{3+} and Tm^{3+} ions, cross-relaxation processes between neighboring Tm^{3+} ions, non-radiative relaxation and phonon-assisted energy transfer[3]. Note that the 455 nm peak which is of interest in this paper increases dramatically its relative intensity compared to the other peaks. This strong non-linearity allows achieving super-resolution in this excitation power region via uSEE microscopy. The spectra are qualitatively similar to the literature reports on similar upconversion particles[4,5].

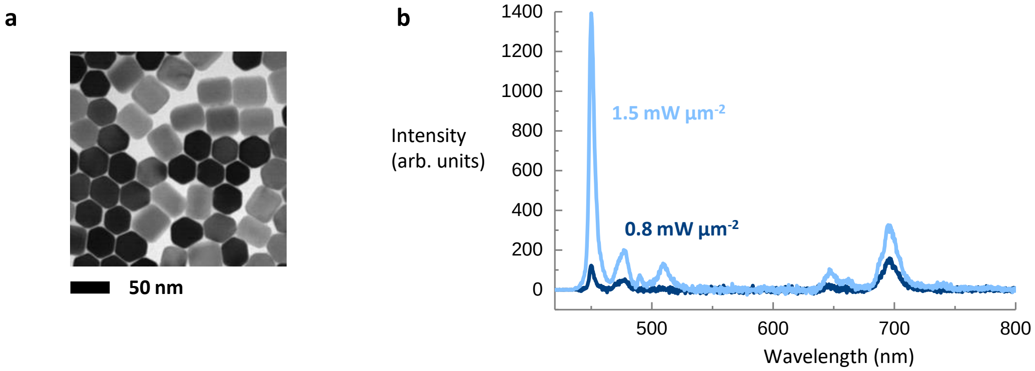

Supplementary Figure 5: **Particle characterization. a** TEM image of the UCNPs. **b** The relative ratio of the luminescent peaks depends on the excitation power.

Setup description

We realize uSEE microscopy on a home-built conventional confocal setup (Supplementary Figure 6). Several additions have been introduced, which are not critical for SEE microscopy, but help to facilitate focusing/sample navigation (optical paths in Supplementary Figure 6 **d**, **e**) and imaging of conventional biologically targeted dyes, such as WGA-Alexa Fluor® 647 (optical path in Supplementary Figure 6 **b**). Additionally, optical path in Supplementary Figure 6 **b** allows STED super-resolution mode for an auxiliary purpose of finding isolated particles. Two laser lines of 976 nm and 808 nm can be used to illuminate the sample. The system allows illumination with individual laser lines, but also simultaneously with the two co-aligned lasers.

976nm laser delivery arm (Supplementary Figure 6 a)

The excitation source is a 976 nm laser diode (Thorlabs, BL976-PAG900), coupled into a polarization maintaining fibre (PM). The output from the PM fibre is collimated by an achromatic doublet lens L_1 (Thorlabs, AC254-035-B, $f = 35$ mm) to a waist of 6 mm. The polarization state of the beam is cleared up by passing the light through a linear polarizer LP (Thorlabs, LPVIS100-MP).

The linearly polarized light passes through a half-wave plate $\lambda_1/2$ (Thorlabs, WPMH10M-980) and a polarising beam splitter (PBS) cube (Thorlabs, CCM1-PBS252/M). The PBS and the half-wave plate tandem enable control of the laser power by rotating the half-wave plate. The part of the beam reflected at the PBS is focused by an achromatic doublet lens L_2 (Thorlabs, AC254-060-B-ML, $f = 60$ mm) onto the power meter (Thorlabs S121C head with PM100D acquisition unit), which is used for active monitoring of the laser power on the excitation beam path.

The part of the beam transmitted through the PBS passes through a set of interchangeable neutral density filters (ND_{set}) (this enables access to a wider range of excitation powers) and the beam is subsequently redirected by two silver mirrors (Thorlabs, PF10-03-P01) towards the rest of the setup.

808nm laser delivery arm (Supplementary Figure 6 **b)**

The 808 nm beam is delivered by a laser diode (Lumics, LU0808M250, 250 mW). The beam is collimated by an air-spaced achromatic doublet lens L_3 (Thorlabs, ACA254-060-B) to the same beam waist as the excitation beam (6 mm).

The beam then passes through a zero-order half-wave plate $\lambda_2/2$ (Thorlabs, WPH10M-808) and a polarising beam splitter (PBS) cube (Thorlabs, CCM1-PBS252/M) to enable control of the optical power passing through the system.

If the STED mode is desired, the beam transmitted through the PBS then passes through a vortex plate VP (Holo/Or, VL-209-5-Y-A). The vortex plate, consisting of 16 discrete phase-steps, transforms the Gaussian beam into a Laguerre-Gaussian (LG) beam with a topological charge $l = 1$.

Afterwards, the laser beam is redirected by a set of two silver mirrors M (Thorlabs, PF10-03-P01) towards the rest of the setup.

Combined delivery path 980nm and 808nm lasers (Supplementary Figure 6 **c)**

A dichroic mirror DM_1 (Chroma, ZT860lpxr) transmits the 976 nm laser and reflects the 808 nm laser to combine and re-direct them towards the rest of the setup. This allows using only one beam at a time, or both co-aligned beams for simultaneous sample illumination.

After the dicroic mirror, the laser beam passes through a position with a removable achromatic doublet lens (Thorlabs, AC254-0400-B-ML, $f = 400$ mm). The RL lens is placed in the beam path only when navigating within the sample. The removable lens (RL) is placed in such a way to generate a partially focused spot at the back-pupil of the microscope objective. This essentially generates a highly defocused beam in the sample plane illuminating a large field-of-view (FOV) improving the navigation within the sample. A slight adjustment to the RL lens positioning displaces the partially focused beam to the outer rim of the back-focal plane of the objective, which allows to image a relatively large FOV in a simplistic dark-field mode. While imaging in the SEE microsopy or STED microscopy mode, the RL lens is not present in the beam path.

After the RL position, the laser beam is reflected by a dichroic mirror DM_2 (Chroma, T750spxrxt) and passes through a removable pellicle RP (Thorlabs, BP145B2). The RP is in place only during sample navigation mode to re-direct the reflected laser light or luminescence towards the camera arms and when acquiring the point spread function (PSF) of the laser(s). Otherwise the RP is removed to maximize the amount of luminescent signal reaching the detector.

Next, the laser beam passes through a quarter-wave plate $\lambda/4$ (Thorlabs, AQWP10M-980), which is oriented in such a way to produce a circularly polarized beam. Afterwards, the beam is reflected by a silver mirror M (Thorlabs, PF10-03-P01) into the rigidly fixed microscope objective MO (Olympus, UPLSAPO 100XO, NA = 1.4, 100x) with a back pupil radius of 4 mm (this leads to a filling factor of $f_0 = 1.5$). Finally, the MO focuses the beam on the sample mounted on a piezo-electric 3-axis stage XYZ (Thorlabs, MAX311D/M with BPC203 piezo controller).

The light reflected, scattered or emitted from the sample is collected through the same objective MO. Then, it can be send to either one of the camera arms for focusing purposes and sample navigation or towards the APD for PSF measurement.

Camera sample navigation - laser reflection/scattering (Supplementary Figure 6 **d)**

We use the laser beams reflected or scattered from the sample for focusing and coarse alignment of the excitation and STED beams with respect to the sample. The faint reflections of the beams are visible whenever the beam encounters an interface with even a very small refractive index mismatch. This helps to focus on a proper position within the sample. Additionally, with the removable lens RL placed in position, we use this beam path to find cells or to find particles in the sample.

The reflected beam propagates back through the microscope objective and is partially reflected on the removable pellicle RP. The removable mirror RM is not in place in this mode and the beam propagates to a fixed pellicle P1 (Thorlabs, BP145B2). Here, it is partially reflected towards the PSF collection arm and partially transmitted towards a second identical pellicle P2 (Thorlabs, BP145B2). At the second pellicle P2 part of the light is transmitted and used for practical alignment purposes (not shown here). The light reflected from P2 passes through a neutral

density filter ND_1 (Thorlabs, NE20A-A). Finally, the reflection from the sample plane is imaged by a tube lens TL_2 (Thorlabs, AC254-200-B-ML, $f = 200\,\mathrm{mm}$) onto a camera CAM2 (CCD camera, Olympus, DP72).

Camera sample navigation - luminescence (Supplementary Figure 6 **e)**

The coarse navigation within a luminescent sample is facilitated by imaging the luminescence onto a second camera – a low-noise camera CAM1 (Thorlabs, DCC3260M). The luminescence is collected through the same objective MO. The removable pellicle RP reflects part of the light reflected, scattered or emitted by the sample. To send the light towards CAM1, a removable silver mirror RM (Thorlabs, PF10-03-P01) is positioned after the pellicle. The luminescent signal is filtered from the excitation light by a shortpass filter F_1 (Thorlabs, FESH0750) and imaged onto the camera by a tube lens TL_1 (Thorlabs, AC254-200-A-ML, $f = 200\,\mathrm{mm}$).

Laser PSF collection pathway (Supplementary Figure 6 **f)**

To measure the PSF of the lasers, we use a standard procedure of imaging the scattered/reflected light by a sample with dispersed gold nanoparticles (size 80 nm) – Supplementary Methods (*Measuring the excitation beam shape using a gold nanoparticle*).

The reflected beam from the particle follows the same trajectory as above until it hits the fixed pellicle P1. At pellicle P1, the beam is reflected towards the PSF collection arm, where it is focused through an achromatic doublet lens L_4 (Thorlabs, AC254-100-B-ML, $f = 100\,\mathrm{mm}$). Afterwards, the beam is reflected at a dichroic mirror DM_3 (Chroma, T750spxrxt) and coupled into multimode fibre MMF (Thorlabs, M43L01, core diameter $105\,\mu\mathrm{m}$).

During the laser PSF measurements, the interchangeable filter IF is not present. Finally, the light exiting the MMF is detected by a single photon avalanche photodiode APD (Excelitas, SPCM-AQRH-14-FC) which is synchronized with the piezo-electric 3-axis stage XYZ (Thorlabs, MAX311D/M with BPC203 piezo controller) to generate the beam PSF profiles.

If this mode is not used, this beam path is closed by a beam block immediately after pellicle P1.

Luminescence PSF collection pathway (Supplementary Figure 6 **g)**

The luminescence is collected through the same objective MO. The removable pellicle RP is removed to maximize the collected luminescent signal. The signal is separated from the laser line by a dichroic mirror DM_2 (Chroma, T750spxrxt) and directed by a set of two silver mirrors M to an achromatic doublet lens L_5 (Thorlabs, AC254-100-A-ML, $f = 100\,\mathrm{mm}$). The signal then passes through a dichroic mirror DM_3 (Chroma, T750spxrxt) and a narrow fluorescence filter IF (Semrock FF01-448/20-25) to isolate the 455 nm emission line. The light is then coupled into a multimode fibre MMF (Thorlabs, M43L01, core diameter $105\,\mu\mathrm{m}$) and detected by a single photon avalanche photodiode APD (Excelitas, SPCM-AQRH-14-FC) which is synchronized with the piezo-electric 3-axis stage XYZ (Thorlabs, MAX311D/M with BPC203 piezo controller) to generate the luminescent image.

For the bio-measurements presented in the paper, the cells are stained with the cellular membrane marker WGA-biotin coupled to streptavidin tagged with Alexa Fluor® 647 and the nuclear intercalating dye DAPI. We visualized the Alexa Fluor® 647 with an identical procedure, where the excitation is done with the 808 nm laser and the detection is through an IF isolating the red emission from the dye (Chroma, ET660/60M-2P).

This pathway is also used to collect spectra of a dense sample. During the spectral acquisition, the fibre collecting the luminescent signal (PSF collection block) is connected to a spectrometer (Ocean Optics USB 2000) rather than an APD. During spectral acquisition, the beam is not scanned but remains static. When not used, this path is blocked after the dicroic mirror DM_2 by a beam block.

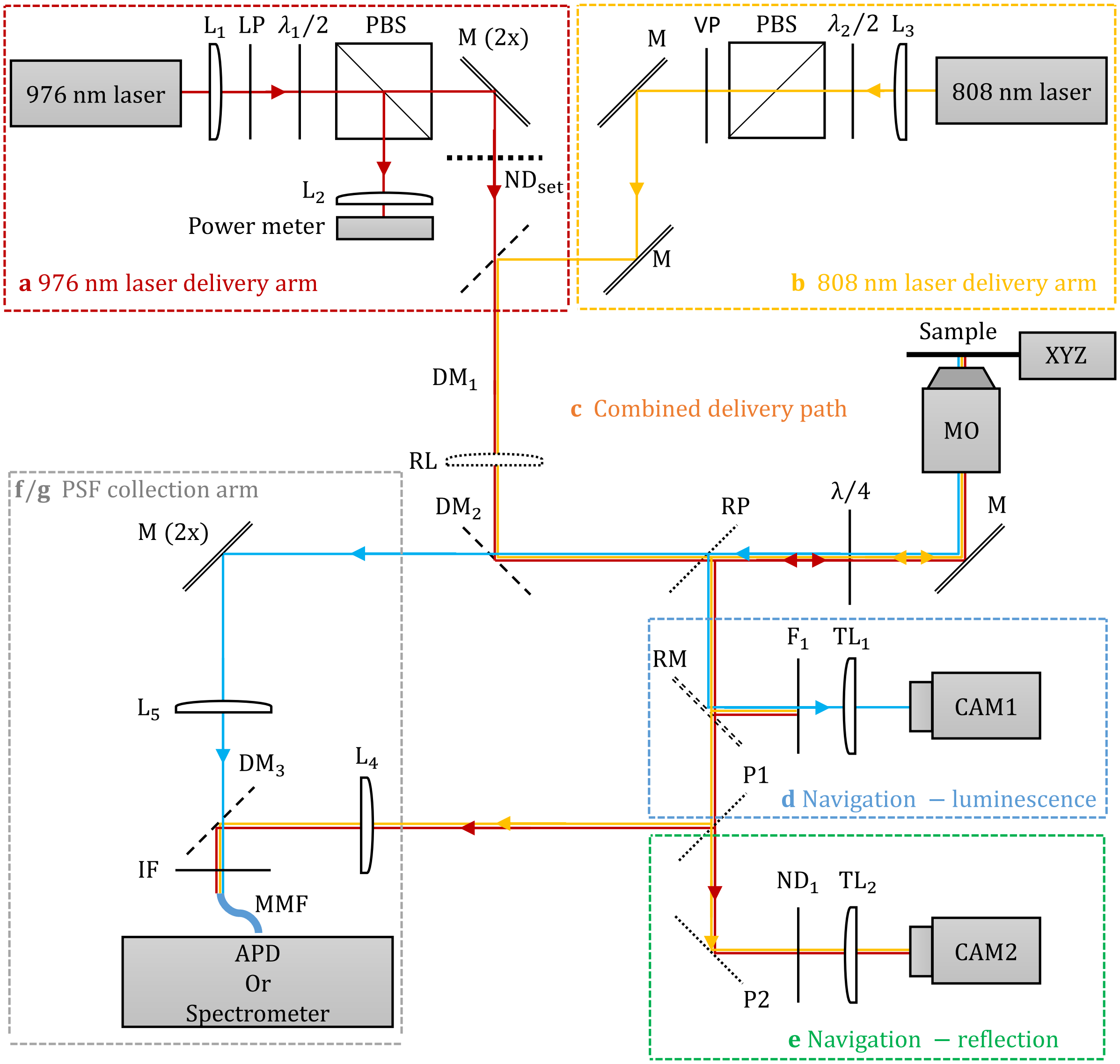

Supplementary Figure 6: **Optical setup**. The sample is positioned on an XYZ piezo stage which is scanned on top of a rigidly fixed microscope objective. It can be illuminated by either of the two lasers of 976 nm and 808 nm or simultaneously by both of them. The light reflected/scattered or emitted from the sample is collected through the same objective MO. The collected light can be sent to either one of the camera arms for focusing purposes and sample navigation or towards a collection multimode fiber MMF which guides the light to an APD for PSF measurement. The fiber can be re-directed towards a spectrometer for spectral measurements. L – lens; LP – linear polarizer; $\lambda/2$ – half-wave plate; $\lambda/4$ – quarter-wave plate; PBS – polarising beam splitter cube; M – mirror; ND – neutral density filter; VP – vortex plate; XYZ – stage; MO – microscope objective; RL – removable lens; TL – tube lens; RM – removable mirror; DM – dichroic mirror; P – pellicle; RP – removable pellicle; CAM – camera; F – filter; IF – interchangeable filter; APD – avalanche photodetector; MMF – multimode fibre.

Measuring the excitation beam shape using a gold nanoparticle

We experimentally measure the excitation beam shape by scanning a gold particle with a diameter of 80 nm through the excitation beam and by detecting the scattered light intensity for each particle position. The scanning of the sample is realized using a commercially available software ImSpec which drives the stage and acquires the particle images. The field-of-view of scanning is set sufficiently large to account for the tails of the excitation beam shape. For all images presented in the paper, we sample above the Nyquist rate to avoid sampling artefacts.

The detector pinhole/fibre diameter is sufficiently large to not influence the excitation beam shape by the detection pathway filtering (Supplementary Note 2, Section F). In our case, the pinhole size/collection fibre diameter is 105 μm and the transverse magnification $M = 76$. This leads to an expected Airy unit in the detection plane equal to 60 μm for $\lambda = 976$ nm wavelength. As a result, the detection pinhole can be considered fully open in the following (even for an out-of-focus gold particle) and the measured beam shape can be considered a valid excitation beam of our optical system.

We verify the above assumption by calculating the excitation beam shape using Eq. (15) and compare the simulation with the experimentally measured excitation beam shape profiles (Supplementary Figure 7 **a**,**b**). In Supplementary Figure 7 **a**, the simulated xz cross-section and the corresponding measurement is presented. Apart from a small tilt of the experimental beam, the two profiles are practically identical. In Supplementary Figure 7 **b**, x and z line cross-sections going through the centre of the beam in Supplementary Figure 7 **a** are presented, with calculated and measured FWHM essentially identical for the two cases.

The FWHM along the lateral (x) and the axial (z) direction is determined from experimental images by using the Matlab function *improfile*. For each direction x and z, we used *improfile* three times to extract pixel values along three adjacent lines. Next, the three *improfile* outputs were averaged to reduce the noise level. Finally, we used Matlab's function *interp1* to increase the sampling rate of the averaged profile ten times to facilitate fitting. We fitted the resulting profile using Matlab's *fit* function with *'smoothingspline'* parameter and with *'SmoothingParam'* set to 0.02. Such fit of the experimental data was subsequently used for the precise determination of the FWHM value.

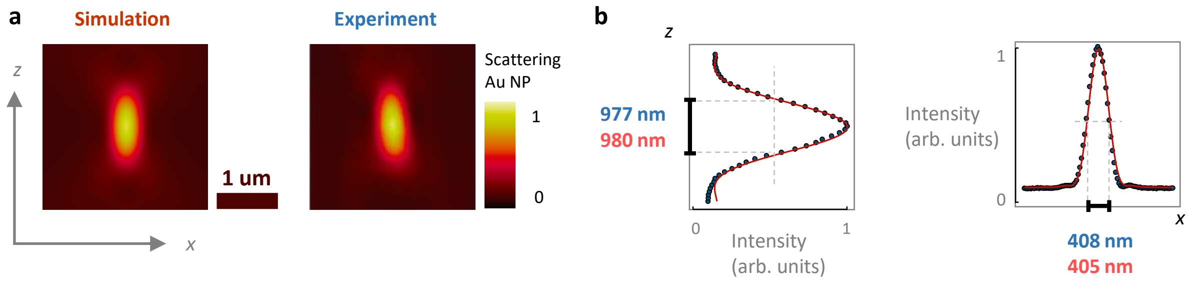

Supplementary Figure 7: **The simulated excitation beam profile is in a good agreement with the experimental one.** **a** Simulated and experimental xz profiles of the excitation beam in the focus of a high NA objective; **b** Line profiles obtained from (**a**) along the z and x axis going through the centre of the beam.

Pre-illumination procedure for UCNPs

As shown in Supplementary Note 1, the UCNP particles we are using show high photo-stability for hours, under relevant imaging conditions of uSEE microscopy and diffraction-limited confocal microscopy. However, to verify the validity of the developed theoretical framework over a broad region of excitation powers, covering different slopes of the excitation-emission curve, we sometimes needed to subject the particle to prolonged periods of intense illumination, outside of typical imaging conditions. Such illumination can lead to a drop of the emission intensity of the particle, which can significantly alter the excitation-emission curve and compromise the presented results.

To avoid such effects, we have employed a pre-illumination procedure. It is illustrated for the same pair of particles as in Supplementary Figure 1 and was applied to the particles after the measurements in Supplementary Figure 1. The UCNP in the middle of the image was centered to the beam and was left there without scanning for a period of 2.5 hours. The excitation power was 2.2 mW at the back focal plane of the objective ($11.8\,\mathrm{mW}\,\mu\mathrm{m}^{-2}$ peak power density at the sample). The emission of the particle declined to about 40% of the initial emission value before stabilizing – Supplementary Figure 8. Evidently from the inset images, this procedure has a local effect and only influences the particle in the middle of the beam. We have used this procedure whenever we wanted to ensure a long-term emission stability under extreme excitation conditions over prolonged periods of time, for example we applied the pre-illumination step to the particle studied in Figure 1 and Figure 3 in the main text. Under typical experimentally relevant conditions, used for obtaining the rest of the data in the paper, this procedure was not needed.

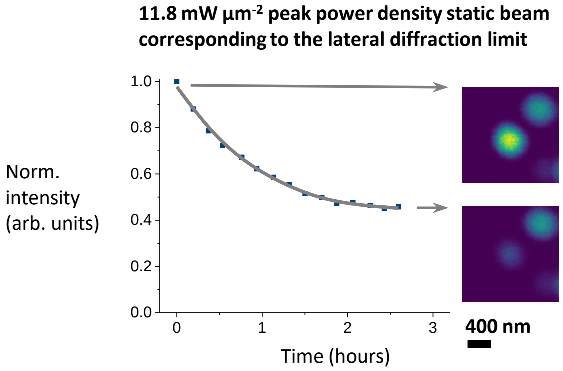

Supplementary Figure 8: **Pre-illuminating procedure**. The pre-illumination procedure reduces the emission intensity of the particle, but allows reaching a stable emission level after illumination at $11.8\,\mathrm{mW}\,\mu\mathrm{m}^{-2}$ peak power density for 2.5 hours.

Measuring the resolution of the system for a non-linear emitter

Experimentally, the resolution of the system for the case of a luminescent particle is obtained by scanning the particle through the excitation beam, acquiring the corresponding image of the particle and measuring the FWHM of the emission profile.

Similar to Supplementary Methods (*Measuring the excitation beam shape using a gold nanoparticle*), the resulting image is used to determine the FWHM along the lateral (x) and the axial (z) direction by using the Matlab function *improfile* averaged over three lines going through the centre of the imaged particle. We again increased the sampling rate of the averaged line profile ten times by using linear interpolation between the original profile points by using *interp1* function in Matlab. We fit the resulting profile averaged over three lines using Matlab's *fit* function with *'smoothingspline'* parameter and with *'SmoothingParam'* set to 0.02. This leads to a sufficiently smooth fit even for highly noisy images at low excitation powers, with very good estimators of the profile's peak and half intensity points – the crucial parameters for determining the FWHM of the non-linear particle. The background level is subtracted from the images to remove bias to the FWHM evaluation.

STED imaging

The stimulated emission depletion (STED) super-resolution modality of the setup is not necessary to achieve uSEE imaging. In our studies we use the STED capability for navigation and verification purposes. STED allows imaging of the UCNPs used in this paper ($NaYF_4$, doped with 20% Yb and 8% Tm) with resolution close to the size of the particles, which helps identifying isolated UCNPs – Supplementary Figure 9 **a**. Additionally, the 808 nm laser line which is used for depletion in the STED mode can be used for excitation of the WGA-Alexa Fluor® 647 dye and help confirm the position of the UCNPs with respect to the cells.

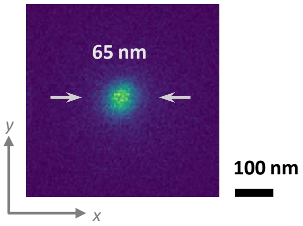

Supplementary Figure 9: **STED image of an upconversion nanoparticle**.

SUPPLEMENTARY REFERENCES

1. Novotny, L. & Hecht, B. *Principles of nano-optics* (Cambridge Univ. Press, Cambridge, 2006).

2. Caillat, L. *et al.* Multiphoton upconversion in rare earth doped nanocrystals for sub-diffractive microscopy. *Applied Physics Letters* **102**, 143114 (2013).

3. Levy, E. S. *et al.* Energy-looping nanoparticles: Harnessing excited-state absorption for deep-tissue imaging. *ACS Nano* **10**, 8423–8433 (2016).

4. Zhan, Q. *et al.* Achieving high-efficiency emission depletion nanoscopy by employing cross relaxation in upconversion nanoparticles. *Nature Communications* **8**, 1058 (2017).

5. Liu, Y. *et al.* Amplified stimulated emission in upconversion nanoparticles for super-resolution nanoscopy. *Nature* **543**, 229–233 (2017).
